# Anti–SARS-CoV-2 Antibody Responses in Convalescent Plasma Donors Are Increased in Hospitalized Patients; Subanalyses of a Phase 2 Clinical Study

**DOI:** 10.3390/microorganisms8121885

**Published:** 2020-11-28

**Authors:** Evangelos Terpos, Marianna Politou, Theodoros N. Sergentanis, Andreas Mentis, Margherita Rosati, Dimitris Stellas, Jenifer Bear, Xintao Hu, Barbara K. Felber, Vassiliki Pappa, Maria Pagoni, Elisavet Grouzi, Stavroula Labropoulou, Ioanna Charitaki, Ioannis Ntanasis-Stathopoulos, Dimitra Moschandreou, Anthi Bouhla, Stylianos Saridakis, Eleni Korompoki, Chara Giatra, Tina Bagratuni, Angelos Pefanis, Sotirios Papageorgiou, Alexandros Spyridonidis, Anastasia Antoniadou, Anastasia Kotanidou, Konstantinos Syrigos, Konstantinos Stamoulis, George Panayiotakopoulos, Sotirios Tsiodras, Leonidas Alexopoulos, Meletios A. Dimopoulos, George N. Pavlakis

**Affiliations:** 1 Department of Clinical Therapeutics, School of Medicine, National and Kapodistrian University of Athens, 11528 Athens, Greece; tsergentanis@yahoo.gr (T.N.S.); j.charitaki@gmail.com (I.C.); johnntanasis@med.uoa.gr (I.N.-S.); e.korompoki@imperial.ac.uk (E.K.); tbagratuni@hotmail.co.uk (T.B.); mdimop@med.uoa.gr (M.A.D.); 2 Hematology Laboratory Blood Bank, Aretaieion Hospital, School of Medicine, National and Kapodistrian University of Athens, 11528 Athens, Greece; mpolitou@med.uoa.gr; 3 Public Health Laboratories, Hellenic Pasteur Institute, 11521 Athens, Greece; mentis@pasteur.gr (A.M.); vlabropoulou@pasteur.gr (S.L.); 4 Human Retrovirus Section, Vaccine Branch, Center for Cancer Research, National Cancer Institute, Frederick, MD 21702-1201, USA; margherita.rosati@nih.gov (M.R.); Dimitrios.stellas@nih.gov (D.S.); george.pavlakis@nih.gov (G.N.P.); 5 Institute of Chemical Biology, National Hellenic Research Foundation, 48 Vassileos Constantinou Ave., 11635 Athens, Greece; 6 Human Retrovirus Pathogenesis Section, Vaccine Branch, Center for Cancer Research, National Cancer Institute, Frederick, MD 21702-1201, USA; jenifer.bear@nih.gov (J.B.); xintao.hu@nih.gov (X.H.); barbara.Felber@nih.gov (B.K.F.); 7 Hematology Unit, Second Department of Internal Medicine, Attikon University General Hospital, School of Medicine, National and Kapodistrian University of Athens, 11528 Athens, Greece; vas_pappa@yahoo.com (V.P.); ampouchla@med.uoa.gr (A.B.); sotirispapageorgiou@hotmail.com (S.P.); 8 BMT Unit, Department of Hematology and Lymphomas, Evangelismos General Hospital, 10676 Athens, Greece; marianpagoni@yahoo.com (M.P.); xgiatra@gmail.com (C.G.); 9 Department of Transfusion Service and Clinical Hemostasis, “Saint Savvas” Oncology Hospital, 11522 Athens, Greece; egrouzi@otenet.gr (E.G.); dimitramos@gmail.com (D.M.); 10 Blood Bank, Evangelismos General Hospital, 10442 Athens, Greece; ssaridak@gmail.com; 11 Department of Internal Medicine, Sotiria General Hospital of Chest Diseases, 11527 Athens, Greece; pefan1@otenet.gr; 12 BMT Unit, University Hospital of Patras, School of Medicine, University of Patras, 26500 Patras, Greece; spyridonidis@upatras.gr; 13 Fourth Department of Internal Medicine, Attikon University General Hospital, School of Medicine, National and Kapodistrian University of Athens, 11527 Athens, Greece; ananto@med.uoa.gr (A.A.); tsiodras@med.uoa.gr (S.T.); 14 First Department of Critical Care Medicine and Pulmonary Services, Evangelismos General Hospital, School of Medicine, National and Kapodistrian University of Athens, 11527 Athens, Greece; akotanid@gmail.com; 15 Oncology Unit, Third Department of Medicine, School of Medicine, National and Kapodistrian University of Athens, 11527 Athens, Greece; ksyrigos@med.uoa.gr; 16 Hellenic National Blood Transfusion Center, 13678 Athens, Greece; kostas.stamoulis@gmail.com; 17 Pharmacology Laboratory, School of Medicine, University of Patras, 26500 Patras, Greece; gpanayiotakopoulos@hotmail.com; 18 National Public Health Organization, 15123 Athens, Greece; 19 Biomedical Systems Laboratory, National Technical University of Athens, 11527 Athens, Greece; leo@mail.ntua.gr

**Keywords:** Covid-19, SARS-CoV-2, novel coronavirus, convalescent plasma, antibodies

## Abstract

We evaluated the antibody responses in 259 potential convalescent plasma donors for Covid-19 patients. Different assays were used: a commercial ELISA detecting antibodies against the recombinant spike protein (S1); a multiplex assay detecting total and specific antibody isotypes against three SARS-CoV-2 antigens (S1, basic nucleocapsid (N) protein and receptor-binding domain (RBD)); and an in-house ELISA detecting antibodies to complete spike, RBD and N in 60 of these donors. Neutralizing antibodies (NAb) were also evaluated in these 60 donors. Analyzed samples were collected at a median time of 62 (14–104) days from the day of first symptoms or positive PCR (for asymptomatic patients). Anti-SARS-CoV-2 antibodies were detected in 88% and 87.8% of donors using the ELISA and the multiplex assay, respectively. The multivariate analysis showed that age ≥50 years (*p* < 0.001) and need for hospitalization (*p* < 0.001) correlated with higher antibody titers, while asymptomatic status (*p* < 0.001) and testing >60 days after symptom onset (*p* = 0.001) correlated with lower titers. Interestingly, pseudotype virus-neutralizing antibodies (PsNAbs) significantly correlated with spike and with RBD antibodies by ELISA. Sera with high PsNAb also showed a strong ability to neutralize active SARS-CoV-2 virus, with hospitalized patients showing higher titers. Therefore, convalescent plasma donors can be selected based on the presence of high RBD antibody titers.

## 1. Introduction

Coronavirus disease 2019 (Covid-19) is an infection caused by the newly discovered severe acute respiratory syndrome coronavirus 2 (SARS-CoV-2), which was first noted in Wuhan, China and spread all over the world [1]. The majority of infected cases have an asymptomatic or mild disease course [2]; however, Covid-19 may become a multisystemic disease with high mortality as several patients develop acute respiratory distress syndrome and need intensive care unit (ICU) support [3,4,5,6].

There are very limited therapeutic options for the management of the disease in severe cases. Among them, remdesivir shortened the time to recovery in adults hospitalized with Covid-19 and evidence of lower respiratory tract infection [7], while low dose dexamethasone resulted in lower 28-day mortality among patients who were receiving either invasive mechanical ventilation or oxygen alone [8]. The use of convalescent plasma showed promising preliminary results [9,10,11,12] and the Food and Drug Administration (FDA) in the USA has provided an emergency use authorization recognizing that the potential benefits outweigh the risks [13], although a recent phase 2 study reported that convalescent plasma did not reduce the risk of progression to severe Covid-19 or the all-cause mortality [14]. The efficacy of this method is based on the accumulating knowledge for the dynamics of immunity against SARS-CoV-2, which is not totally clear to-date. It is important to know the antibody levels at the time of plasmapheresis, whether these levels remain stable compared to the time of screening and their correlation with patient outcomes. A recent study from China showed that all patients who were hospitalized developed antiviral antibodies within 19 days after symptom onset [15], while asymptomatic patients with Covid-19 seemed to have a weaker immune response compared to symptomatic patients [16]. A study from Los Angeles, USA, in 34 patients with mild disease showed a dramatic reduction of recovered patient antibodies within 3 months since the time of infection [9] raising the critical issue of how long these antibodies remain in the body and what is the optimal time frame to be obtained as convalescent plasma for the treatment of patients with severe Covid-19.

The aim of this study was to evaluate the antibody responses in volunteer donors, who participated in a phase 2 trial (NCT04408209) for the use of convalescent plasma for the treatment of severe Covid-19 infection, and to correlate them with clinical characteristics and symptoms of these donors when they suffered from Covid-19.

## 2. Materials and Methods

### 2.1. Study Design

This is an ongoing phase 2 study (NCT04408209) for the use of convalescent plasma for the treatment of severe Covid-19 infection that was started on 28 April 2020, in Greece. This analysis reports the results regarding the presence of anti-SARS-CoV-2 antibodies in volunteer donors who were tested for plasma donation and who consented to further analysis, including evaluation of neutralizing antibodies.

### 2.2. Inclusion Criteria for the Plasma Donors

Main inclusion criteria included: (i) signed informed consent; (ii) confirmed SARS-CoV-2 infection by PCR (methodology in the Supplementary Materials); (iii) interval of at least 14 days after complete recovery from a SARS-CoV-2 infection; and (iv) presence of anti-SARS-CoV-2 antibodies in the testing performed on the day of screening. All inclusion criteria are described in the Supplementary Materials.

### 2.3. Endpoints of the Study Regarding Plasma Donors

(i) titer of anti-SARS-CoV-2 antibodies on the day of screening using a commercial assay; (ii) titer of anti-SARS-CoV-2 antibodies on the day of screening using in-house assays; (ii) titer of neutralizing antibodies against SARS-CoV-2 for those who further consented to give serum sample for this measurement, after protocol amendment; (iii) correlation of the titer of the anti-SARS-CoV-2 antibodies of the donors with age, gender, blood type, presence and type of symptoms, need for hospitalization, need for admission to ICU and need for mechanical ventilation of the donors at the time of their Covid-19 infection. The data collection for plasma donors is fully described in the Supplementary Materials.

### 2.4. Plasma Donors Enrollment

Volunteer donors were tested for the presence of anti-SARS-CoV-2 in the period 28 April 2020, to 30 July 2020, in four centers in Greece. All study procedures were carried out in accordance with the declaration of Helsinki (18th World Medical Association Assembly), its subsequent amendments, the Greek regulations and guidelines, as well as the good clinical practice guidelines (GCP) as defined by the International Conference of Harmonization. The study was also approved by the local ethics committees of all participating hospitals.

### 2.5. Detection of Anti-SARS-CoV2 Antibodies

For the detection of anti-SARS-CoV-2 antibodies, we used three methods; (i) a commercially available ELISA (this was the standard method for making the decision to proceed with plasmapheresis); (ii) an exploratory commercial multiplex serological assay; and (iii) an in-house ELISA, with the latter two assays used for donors who gave consent for all tests.

According to the first method, IgG and IgA anti-SARS-CoV-2 antibodies were detected in the sera of the donors using a semiquantitative commercial ELISA (Euroimmun Medizinische Labordiagnostika AG, Lubeck, Germany), according to the manufacturer (see Supplementary Materials). The method detects antibodies against the recombinant spike protein of the virus (S1 domain) [17].

The ProtATonce multiplex assay (ProtATonce, Athens, Greece) is based on the Luminex^®^ xMAP™ technology to detect total antibodies (IgG/IgM/IgA) and individual antibody isotypes IgG, IgM and IgA against 3 SARS-CoV-2 antigens (S1, like the first method; spike receptor-binding domain (*RBD*) and complete nucleocapsid (*N*) protein. Furthermore, antibodies against human endemic (common-cold) coronaviruses HCoV-OC43 (S1 antigen), HCoV-HKU1 (S1 antigen), HCoV-229E (S1 antigen) and HCoV-NL63 (S1+S2 antigens) were also evaluated. The method is clearly described in https://www.medrxiv.org/content/10.1101/2020.09.09.20191122v2 and briefly in the Supplementary Materials.

An in-house ELISA was also developed to detect either the complete spike (amino acid (AA) 15–1208_2P) or spike RBD (AA 319-525) using mammalian Expi293-cells produced proteins, or *E. coli*-produced nucleocapsid protein spanning the RNA binding domain (AA 47-173; see Supplementary Materials).

### 2.6. SARS-CoV-2 Pseudotype and Live Virus Neutralization Assay

The successive steps of the neutralizing antibody (Nab) assay using SARS-CoV-2 pseudotyped virus [18,19] were as follows: pseudo-virions carrying SARS-CoV-2 spike protein were generated in HEK293 T cells by co-transfecting a plasmid encoding the expression-optimized SARS-CoV-2 spike that lacks 19 C terminal AA and a plasmid encoding Env-defective, luciferase-expressing HIV-1 genome (pHIV_NL_Env-Nanoluc [18,19]). 293T/ACE2wt cells [18,19] were seeded at 15,000 cells per well in a 96-well plate. The next day, eight serial (4-fold) dilutions of heat-inactivated sera (starting at 1:10) were incubated in triplicate with an equal volume of the pseudotyped virions (resulting in 1:20 dilution of serum), and the virion-Ab mixture was used to transduce HEK293T/ACE2wt cells. Two days later, the luciferase levels were measured in the cell extracts, and the ID50 (50% inhibitory dose) was calculated using GraphPad Prism version 8.0 for macOS X (GraphPad Software, Inc., La Jolla, CA, USA) with nonlinear regression curve fit using inhibitor vs. responses variable slope (four parameters).

Neutralization of live CoV-2 virus was performed in Vero 76 clone E6 cells in a BSL-3 facility as described [20]. Briefly, 3-fold serial dilutions of heat-inactivated sera (1:20 to 1:4860) were incubated in duplicate with 30 pfu CoV-2 virus (USA-WA1/2020), and the mixture was used to infect Vero cells. Three days later, the plaques were counted, and plaque reducing neutralization titer (PRNT) compared to the number of plaques obtained with the virus in the absence of Ab (control serum) was calculated.

### 2.7. Statistical Analysis

Descriptive statistics were calculated; serum antibody levels were summarized as the median and interquartile range (IQR), due to the deviation from normality, demonstrated with Shapiro–Wilk test. The results of multi-ELISA and anti-S1–IgG Euroimmun assays were cross-tabulated for the comparative assessment of methods.

At the univariate analysis, the associations between antibody levels and gender, age (<50; ≥50 years), status of symptoms (asymptomatic; symptomatic, no hospitalization; hospitalization), time since symptom onset (<60; ≥60 days) and blood group were evaluated with nonparametric tests (Kruskal–Wallis test, Mann–Whitney-Wilcoxon test for independent samples), Pearson’s chi-squared test or Fisher’s exact test, as appropriate.

At the multivariate analysis, a series of multivariate logistic regression models examined associations between anti-SARS-CoV-2 antibody levels and gender, age, symptoms and time since symptom onset, categorized as detailed above. A total of six separate multivariate logistic regression models were estimated for: anti-S1–IgG–Euroimmun, positivity (cutoff = 1), anti-S1–IgG–Euroimmun (median as the cutoff), anti-N (total, multi-ELISA, median as the cutoff), anti-S1 (total, multi-ELISA, median as the cutoff), anti-RBD (total, multi-ELISA, median as the cutoff) and multi-ELISA positivity, set as dependent variables.

In addition, to further evaluate associations with specific symptoms, multivariate logistic regression analysis examined the associations of the aforementioned six antibody variables with each individual symptom (fever; fatigue; headache; cough; dyspnea; diarrhea; anosmia; taste loss), adjusting for gender, age and time since symptom onset.

Absence of multicollinearity was verified through estimation of condition number and variance inflation factor.

All statistical analyses were performed with STATA/SE version 13 statistical software (Stata Corp., College Station, TX, USA). ELIA AUC, neutralizing Ab ID50 and comparisons were analyzed using GraphPad Prism version 8.0 for macOS X (GraphPad Software, Inc., La Jolla, CA, USA).

## 3. Results

### 3.1. Characteristics of Potential Plasma Donors

Overall, 259 potentially eligible plasma donors were tested for the presence of anti-SARS-CoV-2 antibodies. Among them, 137 (52.8%) were males, whereas 121 (46.7%) were 50 years old or older. At the time of Covid-19 diagnosis, 20 (7.7%) were asymptomatic, 156 (60.2%) were symptomatic but did not need hospitalization and 83 (32%) were hospitalized. Among the 239 symptomatic patients, more than half reported fatigue (n = 143, 59.8%) and fever (n = 131, 54.8%), whereas other commonly reported symptoms included headache (n = 120, 50.2%), anosmia (n = 116, 48.5%), cough (n = 112, 46.8%), and loss of taste (n = 112, 46.8%). The median time from the day of the initial symptoms (or positive PCR assay (PCR+) for those with the asymptomatic disease) until the day of screening was 62 (range: 14–104) days. The characteristics of plasma donors are depicted in Table 1.

### 3.2. Detection of Anti-SARS-CoV-2 Antibodies

The Euroimmun ELISA assay was performed on all potential donors; the ProtATonce multiplex ELISA assay was performed on 213 volunteers, and the in-house ELISA was performed in a subset of 60 volunteers who gave their consent for all the follow-up tests. The Euroimmun ELISA assay is commercially available with a qualitative evaluation, while the multiplex Luminex assay allowed the detection of different antibody types (IgG/IgM/IgA) and individual antibody isotypes. Both assays were performed at a given plasma dilution. In contrast, the in-house ELISA was performed using serial serum dilutions that maximize the accurate determination of Ab levels. The different assays were used to describe our patient cohort.

Anti-SARS-CoV-2 antibodies were detected in 229 (88%) participants with the Euroimmun assay and in 187 (87.8%) with the multiplex assay (based on positivity/negativity described in Supplementary Table S1). Among the 213 participants with both assays performed, 178 (83.6%) had detectable anti-SARS-CoV-2 antibodies with the Euroimmun assay and 187 (87.8%) with the multiplex assay. Importantly, all volunteers with detectable antibodies with the Euroimmun assay, who were also tested by the multiplex assay, had detectable antibodies with the multiplex assay too. Assuming that the anti-S1-IgG Euroimmun is the “gold standard” method, the sensitivity of multi-ELISA is equal to 100%, whereas its specificity is equal to 74.3% (26/35, 95% CI: 56.7–87.5%) (Table 2).

### 3.3. Correlations of Anti-SARS-CoV-2 Antibody Titer with Clinical Features

**Figure 1:**
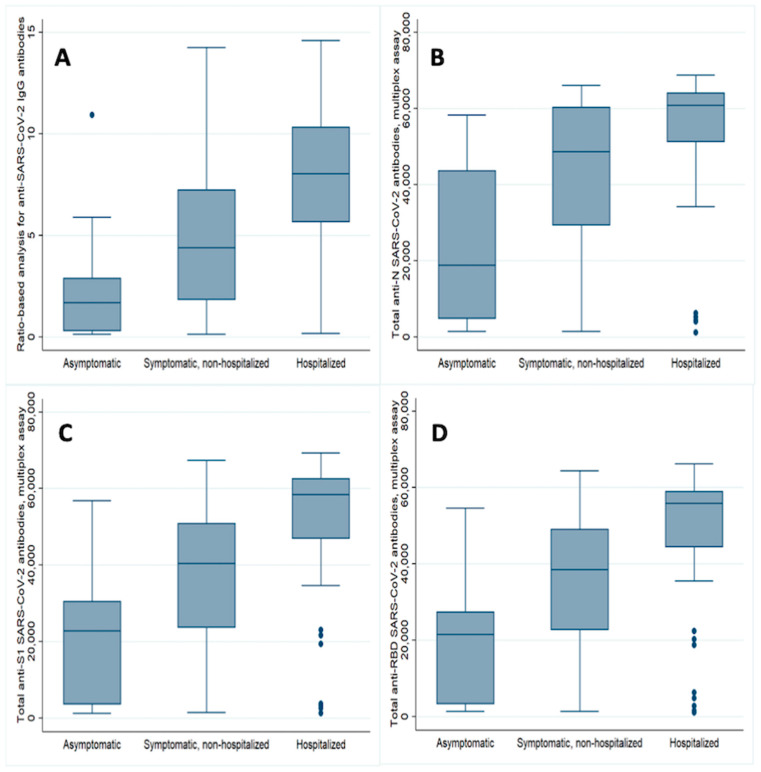
Convalescent plasma donors anti-SARS-CoV-2 antibodies against all studied epitopes on the day of screening, according to disease severity (**A**–**D**). The median ratio (IQR) for anti-S1-IgG antibodies using the Euroimmun ELISA method were as follows: 1.69 (2.59) vs. 4.40 (5.42) vs. 8.04 (4.69) for asymptomatic, non-hospitalized symptomatic and hospitalized symptomatic, respectively (*p* = 0.0001, Kruskal–Wallis test); (**A**) the respective values of the median fluorescent intensity (IQR) for the total (IgM+IgA+IgG) antibodies, using the multiplex assay, against basic nucleocapsid (N) protein were: 18,875 (38,803) vs. 48,703 (30,981) vs. 60,913 (12,810) (*p* = 0.0001; (**B**) while against S1 were 22,806 (26,735) vs. 40,436 (27,514) vs. 58,377 (15,733) (*p* = 0.002)); (**C**) and against receptor-binding domain (RBD) were 21,515 (24,077) vs. 38,556 (26,393) vs. 55,869 (14,522) (*p* = 0.002; (**D**)).

Potential donors who had asymptomatic Covid-19 had lower antibody titer compared to those who had the symptomatic disease but did not need hospitalization or those who were hospitalized (Figure 1). The results were consistent across all the epitopes examined with both the Euroimmun Elisa and the ProtATonce multiplex assay (Table 3; Figure 1).

**Figure 2:**
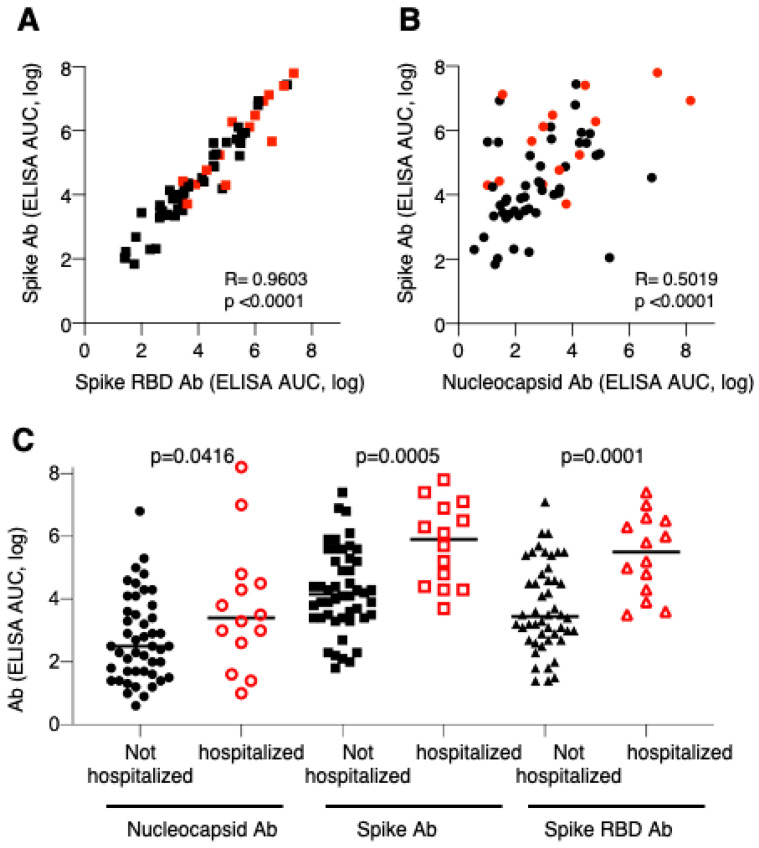
Correlation of CoV-2 antibody levels. Binding Ab levels were measured by ELISA using serially diluted sera and were expressed as area-under-the-curve (AUC). Correlation of spike and spike RBD (**A**) and spike and nucleocapsid (**B**) are shown. (**C**) Comparison of nucleocapsid, spike and spike RBD antibody levels (AUC, log) in non-hospitalized and hospitalized Covid patients. *p* values are from unpaired *t*-tests. Red symbols denote sera from hospitalized Covid patients.

Samples from 60 consented patients, who gave their written consent for more blood to be taken, were also analyzed by an in-house ELISA measuring antibodies to complete spike protein, spike RBD and nucleocapsid using serial serum dilutions. After analysis of serially diluted samples, the antibody levels were expressed as area-under-the-curve (AUC) values that allows more accurate determination of their magnitude. Comparison of the spike and spike-RBD AUC values showed excellent correlation (Pearson’s R = 0.96, *p* < 0.001), supporting the strong recognition of the SARS-CoV-2 induced antibodies of RBD within the spike protein (Figure 2A). The ELISA analysis further showed that hospitalized patients had overall higher spike antibody levels in agreement with the data shown in Figure 1 (Figure 2A,C). There is also a correlation of spike and nucleocapsid antibody responses, although to a lesser extend (Pearson’s R = 0.5, *p* < 0.001; Figure 2B), which further showed that some patients have high spike antibodies but low N antibodies and vice versa, with hospitalized patients showing high responses to both antigens. Comparison of nucleocapsid antibody responses showed slightly higher levels in hospitalized Covid patients (Figure 2C), while the spike and spike-RBD levels were greatly increased using the quantitative ELISA assay.

Significantly less volunteers <50 years had detectable anti-SARS-CoV-2 antibodies with both Euroimmun and multiplex assay compared with those aged 50 years and above (positivity rates: Euroimmun method, 77.4% vs. 90%, respectively, *p* = 0.007; multiplex assay, 82.5% vs. 92.9%, *p* = 0.011). Donors <50 years had lower antibody titer compared with older patients, while those who were tested within 60 days from the first day of symptoms or PCR+ for the asymptomatic disease had higher antibody titer (Table 3). No differences regarding anti-SARS-CoV-2 antibody production were shown among donors with distinct blood types.

The multivariate analysis regarding the results derived from both the Euroimmun and the multiplex assays showed that age ≥50 years and need for hospitalization correlated with higher antibody titers, while asymptomatic status correlated with lower antibody titers (Table 4). Similar results were obtained when age was alternatively treated as a continuous variable. In the multivariate logistic regression analysis examining associations between individual symptoms and antibody levels, based on both assays, there was an independent correlation between anti-SARS-CoV-2 antibodies with anosmia and loss of taste (Table 5).

### 3.4. Presence of anti-SARS-CoV-2 Neutralizing Antibodies (NAbs)

**Figure 3:**
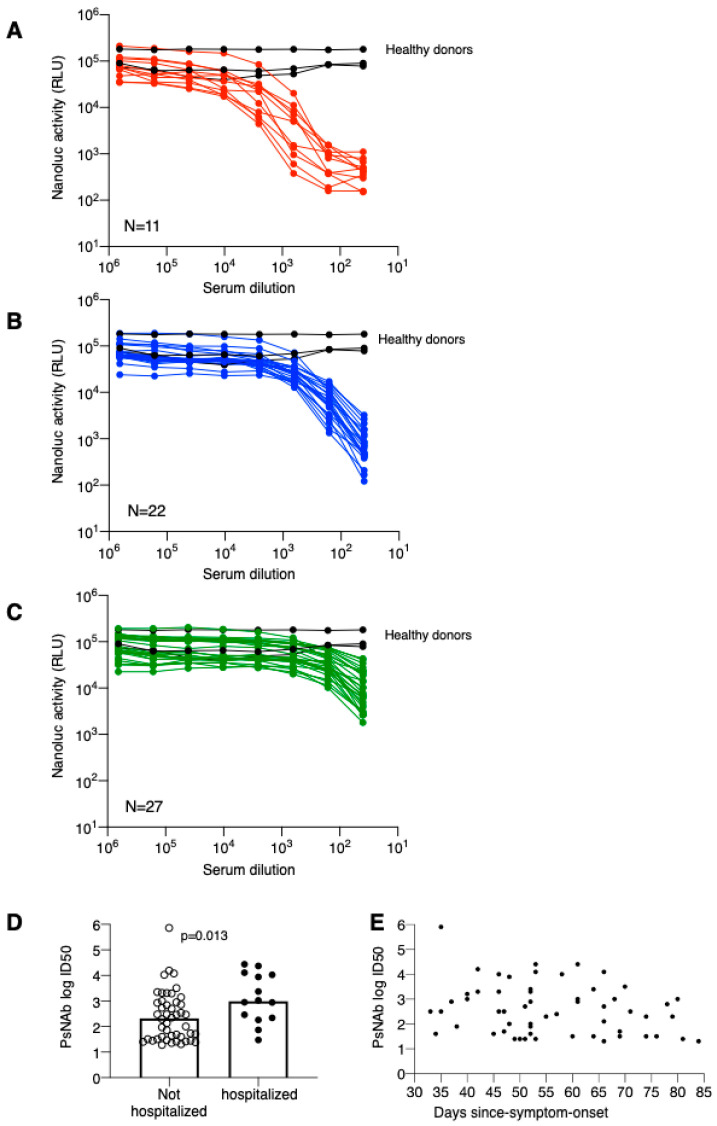
Neutralization curves of individual sera were measured using pseudotype virus assay. The data are plotted according to neutralization strength with high PsNAb (**A**), medium PsNAb (**B**) and low PsNAb (**C**). Black curves denote sera from healthy donors. (**D**) Comparison of pseudotype virus PsNAb ID50 in not hospitalized and hospitalized patients. *p* value is from an unpaired nonparametric *t*-test. (**E**) Pseudotype virus PsNAb ID50 plotted as measured days since symptom onset.

Overall, 60 patients provided their consent for supplemental blood sampling, at the same time points as described previously, in order to evaluate the presence of NAbs against the novel coronavirus SARS-CoV-2. Neutralization capability of serially diluted sera was measured against an HIV-derived pseudotyped virus (Figure 3). The neutralization data were grouped into sera showing high (N = 11; 18%; Figure 3A), medium (N = 22, 45%; Figure 3B) and low (N = 27, 37%; Figure 3C) levels. Comparison of individual ID50 PsNAb titers showed significantly higher titers in hospitalized patients (Figure 3D). On the other hand, the magnitude of PsNAb titers did not correlate to time-since-symptom-onset (day 33–84) (Figure 3E) in this cohort of Covid-19 patients. Thus, the severity of Covid-19 disease is key in determining spike Ab and NAb levels.

**Figure 4:**
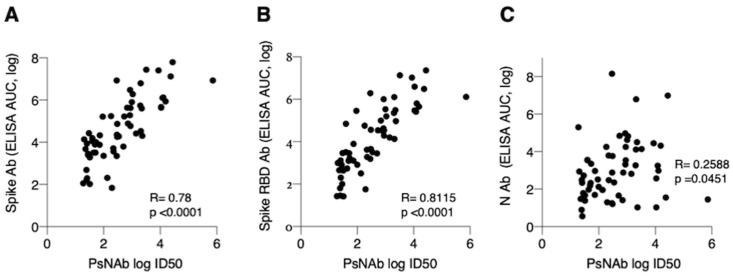
Correlation of PsNAb to Ab measured by ELISA assays. Correlation of ID50 NAb and (**A**) spike (r = 0.78, *p* < 0.0001), (**B**) spike RBD (r = 0.81, *p* < 0.0001) and (**C**) nucleocapsid (*r* = 0.26, *p* = 0.05) AUC measured by ELISA.

Importantly, NAb ID50 titers significantly correlated with the complete spike AUC values (Pearson’s r = 0.78, 95% CI: 0.66 to 0.86, *p* < 0.0001) (Figure 4A) and spike RBD AUC values (Pearson’s r = 0.81, 95% CI: 0.70 to 0.88, *p* < 0.0001) (Figure 4B), and only a marginal association emerged with the nucleocapsid AUC values (Pearson’s r = 0.26, 95% CI: 0.01 to 0.48, *p* = 0.05) (Figure 4C).

**Figure 5:**
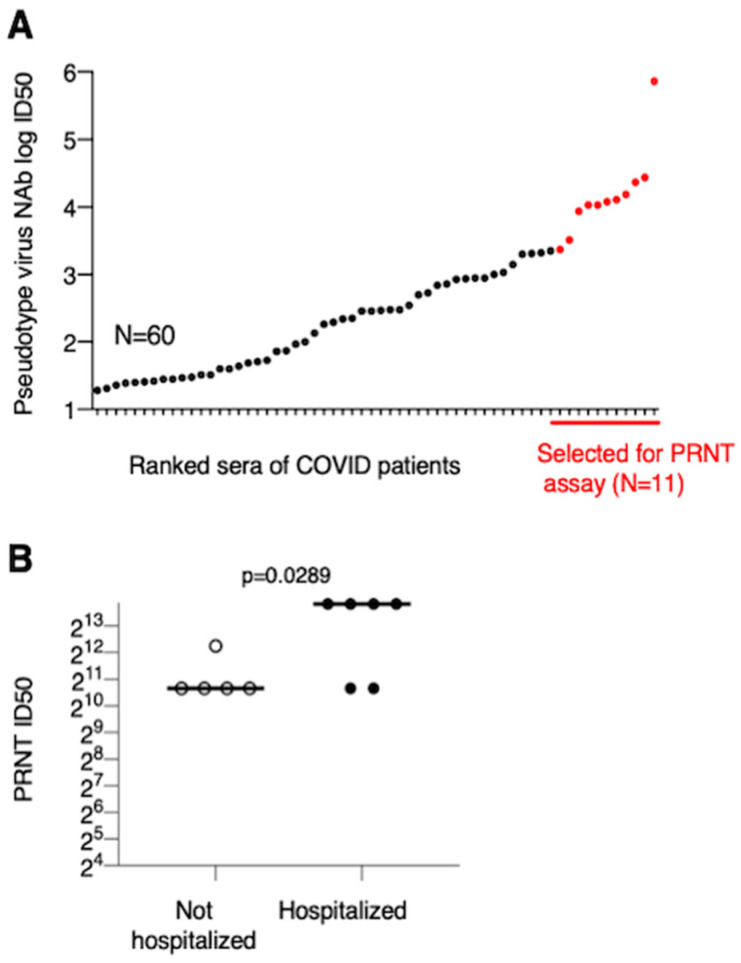
Live-virus neutralization by sera with high pseudotype virus NAb titers. (**A**) The ranked log ID50 values from 60 sera measured at screening are plotted. Eleven sera with the highest PsNAb are marked in red. (**B**) Live-virus neutralization measured as PRNT ID50 values (log2 transformed) are compared from not hospitalized and hospitalized patients. PRNT ID50 > 4860 are plotted as 14,580, reflecting the next dilution. *P* value is from an unpaired parametric *t*-test.

Pseudotype NAb ID50 values were plotted by ranking the sera of the Covid patients (Figure 5A). A selected subgroup of 11 sera with the highest PsNAb titers (indicated in Figure 5A) was subjected to live CoV-2 virus neutralization by measuring the plaque reducing neutralization titer (PRNT) in Vero cells. In this assay, 100% of convalescent sera were able to potently neutralize live CoV-2 virus with ID50 titers ranging from 1:1650 to >1:4860. The sera from hospitalized patients showed significantly higher PRNT ID50 titers (Figure 5B).

Our data show a strong correlation of spike Ab titer and neutralization capability; sera with strong pseudotype virus neutralization capability are also able to potently neutralize active SARS-CoV-2 virus.

## 4. Discussion

Elucidating the kinetics of humoral immune response in SARS-CoV-2 infection is of high priority because it will help us draw conclusions on immunity and its applications regarding population screening studies, convalescent plasma collection [21] and the assessment of the vaccine immunogenicity. In our study, we measured antibodies against SARS-CoV-2 in potential convalescent plasma donors as part of a phase 2 clinical trial assessing the efficacy of convalescent plasma in treating Covid-19 infection. We screened 259 candidate plasma donors, 74 of whom proceeded to plasmapheresis according to the predefined criteria. We assessed humoral immune response with both a commercially available ELISA, which detects antibodies against the recombinant S spike protein of SARS-CoV-2, and a ProtATonce multiplex ELISA, which detects IgG and IgM antibodies against S1, N and RBD epitopes of the novel coronavirus.

We found that symptomatic patients had higher levels of antibodies. We confirmed a previous finding that asymptomatic donors had lower antibody titers compared to symptomatic, and this is in agreement with previous studies that recruited 34 and 37 patients, respectively [9,16]. The previous studies have examined the development of IgG antibodies against either SARS-CoV-2 spike receptor-binding domain or against antigens containing the nucleoprotein and a peptide from the spike protein of SARS-CoV-2 epitopes. In comparison, lower levels of IgG antibodies against S protein and total antibodies against S protein, N protein and RBD protein were recorded in our study. As became also evident in our study, a stratification of antibody levels according to the disease severity seems to emerge since patients who needed hospitalization had higher antibody levels than symptomatic outpatients. This is also in line with the results of a study, including 59 recovered patients who were evaluated for the presence of neutralizing antibodies. Patients with severe Covid-19 disease showed the highest titers of neutralizing antibodies among patients with severe, moderate, mild or asymptomatic disease. Interestingly, asymptomatic patients did not show an adequate immune response in terms of neutralizing antibodies [22]. However, in another study, the need for intensive care unit support among hospitalized patients with Covid-19 did not predict higher titers of anti-SARS-CoV-2 IgG RBD or anti-S neutralizing antibodies [23]. It has not been determined whether the more severe disease can elicit stronger immune responses or, inversely, a strong immune response may lead to the hyperactivation of immune cells and trigger a cytokine storm, which in turn may result in ARDS and/or multiple organ failure characterizing the critically ill Covid-19 patients [24].

The antibody titers were lower in younger Covid-19 patients (<50 years), a finding that persisted in the multivariate analysis implying that age can be a predictor of immune response irrespective of the disease severity. Several factors can influence the strength of humoral response to either infection or vaccination, but it is well documented that genetic variants and age can be strong predictors of humoral immune responses [25]. The fact that this difference was shown for antibodies against all epitopes under investigation may exclude the possibility of interfering cross-reactivity from previous coronavirus infection in the measured antibodies titers in older patients. Our findings are in agreement with previous studies showing that older patients had significantly higher plasma IgG and neutralizing antibody titers than young patients [17,26]. It has been supported that a more intense inflammatory status and a defective T-cell response among older patients may trigger a more potent anti-SARS-CoV-2 antibody production compared to younger ones [26].

In our study, the antibody titers were lower in patients whom we screened after 60 days from the onset of symptoms, both in the univariate and multivariate analyses. This implies that the time from disease onset to antibody evaluation has an impact on the detected titers of anti-SARS-CoV-2 antibodies. This is consistent with previous studies showing that antibody levels decrease over time [9,16,19,27].

A finding that was previously published by this study was the decrease in antibody titers between the time of screening and the time of plasmapheresis [21]. This is also described in other studies [28]. Taking into account that we performed screening at a median of 62 days after symptoms onset or PCR+ (for asymptomatic infection), a decrease in the antibody titers seems to have occurred as early as after two months. However, the rate of decline may vary significantly among patients. Data on the strength and duration of humoral immunity against SARS-CoV-2 is still sparse. A number of reports analyzing the dynamics of antibody response during SARS-CoV-2 infection suggest that IgM seroconversion can take place at a median of 12 days from infection, whereas IgG antibody levels rise gradually from week 3 to week 7 [28]. Weaker or delayed responses may be seen especially in patients with mild disease. Asymptomatic patients may not have detectable IgG antibodies after 3–4 weeks after the onset of symptoms, and antibody levels start to decline rapidly during the “early convalescent phase” [16]. Furthermore, a study including 34 patients with Covid-19 has reported a half-life of approximately 36 days during the observation period for the IgG antibodies against spike receptor-binding domain in asymptomatic patients [9]. From our cohort of 74 patients who underwent plasmapheresis, only 2 (2.7%) were asymptomatic. Thus we suggest that the observed decline of antibodies 1.8 to 3.5 months post Covid-19 diagnosis also applies to symptomatic patients. Another report has shown that 4/8 convalescent patients with Covid-19 had decreased neutralizing antibodies 6–7 weeks after symptom onset [17]. In line with these findings, it has been recently reported that both IgG levels and neutralizing antibodies from SARS-CoV-2 patients start to decrease 2–3 months after the infection [16]. Variations observed among studies could be attributed to differences in the sensitivity and specificity of the methods used, along with differences in the type of the examined epitope. Furthermore, since it is difficult to make comparisons across studies and extrapolate the results, longitudinal studies are needed in order to confirm the dynamic pattern of anti-SARS-CoV-2 antibodies, including the rate of decline after reaching a plateau and assess the duration of seropositivity according to different disease severity. Regarding other known coronaviruses, the antibodies against SARS-CoV-1 and MERS-CoV seem to be detectable up to 2 and approximately 3 years, respectively [13,29].

Differences in the profile of the antibody response across patients may reveal important aspects of the Covid-19 spectrum of clinical manifestations. In our study, we consistently found a correlation of anti-S antibodies or RBD plus S or N with fever, anosmia and taste loss. spike S protein mediates virus entry into the cell. The significant correlation in the multivariate analysis of symptoms related to the neural system, such as headache and anosmia, may highlight the fact that the SARS-CoV-2 virus employs protein S1, which enables the virion to adhere to the cell membrane by interacting with the host ACE2 receptor [30]. ACE2 is a functional receptor for SARS-CoV-2, and its expression in the nervous system suggests that the virus can cause neurological symptoms [5,31]. The correlation of anti-SARS-CoV-2 antibodies with clinical manifestations may also reflect the previously discussed association of humoral immune response with the disease severity.

Moreover, we did not find any association between antibody production and blood type. This is also consistent with previous reports in the field [27]. Several studies have shown that blood type is not a prognostic factor for dismal outcomes among patients with symptomatic Covid-19 disease [32]. However, blood type 0 seems to confer protection against SARS-CoV-2 infection compared to other blood types, probably by preventing the viral entry into host cells [32,33].

Interestingly, we found that complete spike and spike RBD ELISA measurements significantly correlated with the PsNAb assay measurements. Importantly, we also showed potent neutralization of live CoV-2 virus. Therefore, plasmapheresis should be performed in people with known high spike RBD antibody titers and, consequently, high NAb titers in order to optimize efficacy in convalescent plasma recipients. Usually, hospitalized patients with severe or moderate symptoms have higher antibody titers. In this context, this population needs to be approached before hospital discharge and asked to participate in pertinent clinical studies by providing permission to use their existing biological samples for research (anonymously), being screened for RBD antibody levels and NAb after hospital discharge; and agree to undergo plasmapheresis, if their RBD Ab titers are above a certain threshold. Another target population could be symptomatic but non-hospitalized patients in the community.

Among the limitations of our study, we should note that the evaluation of antibody production was a secondary study endpoint in the context of the phase 2 clinical trial of convalescent plasma. Therefore, it may be underpowered in terms of highlighting significant associations in subgroup analyses. Furthermore, multiple serial assessments of antibody status would provide a more accurate evaluation of the longitudinal dynamics of the humoral response against the novel coronavirus SARS-CoV-2.

## 5. Conclusions

In conclusion, lower anti-SARS-CoV-2 antibody titers against all studied epitopes were found in asymptomatic patients, in patients younger than 50 years and in those who were tested 60 days or more after the onset of symptoms. Sera with high PsNAb also showed a strong ability to neutralize active SARS-CoV-2 virus, with hospitalized patients showing higher titers. Therefore, convalescent plasma donors can be selected based on the presence of high RBD antibody titers. Further research is needed to determine the kinetics of NAbs concentration and the impact on host immunity against SARS-CoV-2.

## Figures and Tables

**Table 1:** Characteristics of donors on the day of screening and on the day of plasmapheresis.

	Date of Screening, n = 259 (%)	Date of Plasmapheresis, n = 74 (%)
Gender		
*Female*	122 (47.1)	31 (41.9)
*Male*	137 (52.8)	43 (58.1)
Age (years)		
*<50*	138 (53.2)	44 (59.5)
*≥50*	121 (46.7)	30 (40.5)
Symptoms		
*Asymptomatic*	20 (7.7)	2 (2.7)
*Symptomatic, no hospitalization*	156 (60.2)	53 (71.6)
*Hospitalization*	83 (32.0)	19 (25.7)
*Fever*	131 (50.5)	36 (48.7)
*Fatigue*	143 (55.2)	37 (50.0)
*Headache*	120 (46.3)	43 (58.1)
*Cough*	112 (43.2)	29 (39.2)
*Dyspnea*	72 (27.7)	25 (33.8)
*Anosmia*	116 (44.7)	41 (55.4)
*Taste loss*	112 (43.2)	32 (43.2)
Time since symptom onset (days)		
<60	116 (44.7)	49 (66.2)
≥60	143 (55.2)	25 (33.8)
Blood group ^§^		
*0*	56 (44.8)	33 (44.5)
*A*	49 (39.2)	30 (30.5)
*B*	14 (11.2)	8 (10.8)
*AB*	6 (4.8)	1 (1.3)

^§^ available in a subcohort of 125 patients.

**Table 2:** Comparative evaluation of results derived from the Multi-ELISA and anti-S1–IgG Euroimmun assays.

	Anti-S1–IgG Euroimmun (−)	Anti-S1–IgG Euroimmun (+)	Total
Multi-ELISA (−)	26	0	26
Multi-ELISA (+)	9	178	187
Total	35	178	213

**Table 3:** Results of the univariate analysis examining associations between anti-SARS-CoV-2 antibody levels and demographic-clinical variables. Multi-ELISA was performed in 213 of 259 patients in the cohort. Bold cells denote statistically significant associations.

	Anti-S1–IgG–Euroimmun		Anti-S1–IgG–Euroimmun		Anti-N (Total, Multi-ELISA)		Anti-S1 (Total, Multi-ELISA)		Anti-RBD (Total, Multi-ELISA)		Multi-ELISA	
Variables	Median OD (IQR)	*p*	Positivity rate (%)	*p*	Median MFI (IQR)	*p*	Median MFI (IQR)	*p*	Median MFI (IQR)	*p*	Positivity rate (%)	*p*
Gender		0.036 ^M^		0.810 ^C^		0.560 ^M^		0.087 ^M^		0.044 ^M^		0.663 ^C^
*Female*	4.27 (6.21)		83.6% (102/122)		53,886 (33,610)		41,514 (31,155)		39,398 (30,705)		86.7% (85/98)	
*Male*	6.07 (6.36)		82.5% (113/137)		53,799 (27,084)		46,282 (27,372)		46,097 (28,168)		88.7% (102/115)	
Age (years)		<0.0001 ^M^		0.007 ^C^		<0.0001 ^M^		<0.0001 ^M^		<0.0001 ^M^		0.011 ^C^
<*50*	3.94 (5.10)		77.4% (106/137)		44,350 (39,814)		35,294 (31,707)		33,714 (32,379)		82.5% (94/114)	
*≥50*	7.34 (6.16)		90.0% (108/120)		60,060 (14,067)		54,768 (18,621)		52,254 (19,002)		92.9% (93/99)	
Symptoms		0.0001 ^KW^		0.001 ^C^		0.0001 ^KW^		0.002 ^KW^		0.002 ^KW^		0.002 ^C^
*Asymptomatic*	1.69 (2.59)		55.6% (10/18)		18,875 (38,803)		22,806 (26,735)		21,515 (24,077)		54.6% (6/11)	
*Symptomatic, no hospitalization*	4.40 (5.42)		82.1% (128/156)		48,703 (30,981)		40,436 (27,154)		38,556 (26,393)		88.0% (117/133)	
*Hospitalization*	8.04 (4.69)		91.6% (76/83)		60,913 (12,810)		58,377 (15,733)		55,869 (14,522)		92.7% (63/68)	
Time since symptom onset (days)		0.024 ^M^		0.401 ^C^		0.865 ^M^		0.298 ^M^		0.306 ^M^		0.082 ^C^
<*60*	6.09 (6.52)		85.3% (93/109)		54,381 (24,571)		46,547 (25,020)		45,849 (25,130)		92.0% (92/100)	
*≥60*	4.68 (6.12)		81.3% (113/139)		53,672 (34,210)		42,246 (33,656)		41,566 (33,483)		84.1% (90/107)	
Blood group ^§^		0.149 ^KW^		0.323 ^F^		0.566 ^KW^		0.124 ^KW^		0.159 ^KW^		0.102 ^F^
*0*	5.98 (7.59)		87.5% (49/56)		53,840 (24,779)		50,778 (30,924)		50,069 (30,947)		92.6% (50/54)	
*A*	4.53 (6.13)		79.6% (39/49)		53,736 (34,412)		41,571 (36,020)		38,441 (32,382)		81.3% (39/48)	
*B*	4.07 (5.60)		71.4% (10/14)		50,069 (54,676)		42,546 (35,492)		41,283 (50,078)		71.4% (10/14)	
*AB*	8.30 (3.88)		100.0% (6/6)		55,090 (11,531)		59,310 (16,950)		50,144 (16,903)		100% (5/5)	

^§^ available in a subcohort of 125 patients; ^C^: Pearson’s chi-squared test; ^F^: Fisher’s exact test; ^KW^: Kruskal–Wallis test, ^M^: Mann–Whitney-Wilcoxon test for independent samples; MFI: mean fluorescence intensity; OD: optical density.

**Table 4:** Results of the multivariate logistic regression analysis examining associations between anti-SARS-CoV-2 antibody levels and demographic-clinical variables. Bold cells denote statistically significant associations.

	Anti-S1–IgG–Euroimmun, Positivity (Cutoff = 1)		Anti-S1–IgG–Euroimmun, Median OD as the Cutoff ^§^		Anti-N (Total, Multi-ELISA), Median MFI as the Cutoff ^§^		Anti-S1 (Total, Multi-ELISA), Median MFI as the Cutoff ^§^		Anti-RBD (Total, Multi-ELISA), Median MFI as the Cutoff ^§^		Multi-ELISA Positivity	
Variables	OR (95% CI)	*p*	OR (95% CI)	*p*	OR (95% CI)	*p*	OR (95% CI)	*p*	OR (95% CI)	*p*	OR (95% CI)	*p*
Gender												
*Female*	Ref.		Ref.		Ref.		Ref.		Ref.		Ref.	
*Male*	0.98 (0.47–2.02)	0.951	1.45 (0.82–2.58)	0.200	0.73 (0.39–1.38)	0.332	1.31 (0.69–2.47)	0.408	1.45 (0.78–2.69)	0.240	1.21 (0.49–3.02)	0.681
Age (years)												
<*50*	Ref.		Ref.		Ref.		Ref.		Ref.		Ref.	
*≥50*	2.85 (1.24–6.55)	0.014	2.88 (1.60–5.18)	<0.001	5.83 (3.06–11.11)	<0.001	4.30 (2.27–8.13)	<0.001	3.56 (1.91–6.65)	<0.001	4.28 (1.38–13.24)	0.012
Symptoms												
*Asymptomatic*	0.07 (0.01–0.29)	<0.001	0.10 (0.01–0.82)	0.033	0.18 (0.02–1.69)	0.133	0.53 (0.09–3.09)	0.479	0.49 (0.08–2.82)	0.423	0.06 (0.01–0.35)	0.002
*Symptomatic, no hospitalization*	Ref.		Ref.		Ref.		Ref.		Ref.		Ref.	
*Hospitalization*	1.79 (0.71–4.49)	0.214	4.11 (2.13–7.90)	<0.001	2.64 (1.32–5.30)	0.006	4.78 (2.31–9.86)	<0.001	3.79 (1.88–7.64)	<0.001	1.22 (0.40–3.71)	0.722
Time since symptom onset (days)												
<*60*	Ref.		Ref.		Ref.		Ref.		Ref.		Ref.	
*≥60*	0.62 (0.29–1.31)	0.207	0.36 (0.20–0.66)	0.001	0.77 (0.41–1.44)	0.415	0.56 (0.30–1.06)	0.077	0.53 (0.29–0.99)	0.048	0.38 (0.14–1.01)	0.052

^§^ In these logistic regression models, serum antibody levels were converted to a binary dependent variable, based on the median value of the sample (0: ≤median, 1: >median). Median values were: 5.47 for anti-S1–IgG–Euroimmun; 53,799 for anti-N; 45,036 for anti-S1 (multi-ELISA); and 42,753 for anti-RBD (multi-ELISA). MFI: mean fluorescence intensity; OD: optical density.

**Table 5:** Results of the multivariate logistic regression analysis examining associations between individual symptoms and anti-SARS-CoV-2 antibody levels. All logistic regression models were adjusted for gender, age and time since symptom onset. Bold cells denote statistically significant associations.

	Anti-S1–IgG–Euroimmun, Positivity (cutoff = 1)		Anti-S1–IgG–Euroimmun, Median OD as the Cutoff ^§^		Anti-N (Total, Multi-ELISA), Median MFI as the Cutoff ^§^		Anti-S1 (Total, Multi-ELISA), Median MFI as the Cutoff ^§^		Anti-RBD (Total, Multi-ELISA), Median MFI as the Cutoff ^§^		Multi-ELISA Positivity	
Symptoms	OR (95% CI)	*p*	OR (95% CI)	*p*	OR (95% CI)	*p*	OR (95% CI)	*p*	OR (95% CI)	*p*	OR (95% CI)	*p*
Fever	4.25 (1.90–9.51)	<0.001	3.14 (1.76–5.60)	<0.001	1.58 (0.84–2.97)	0.152	2.72 (1.45–5.10)	0.002	2.49 (1.34–4.62)	0.004	3.93 (1.43–10.80)	0.008
Fatigue	1.44 (0.70–2.97)	0.323	1.23 (0.70–2.16)	0.482	0.74 (0.40–1.39)	0.353	1.27 (0.68–2.35)	0.453	1.15 (0.63–2.12)	0.646	1.19 (0.49–2.88)	0.694
Headache	2.34 (1.09–5.03)	0.029	1.00 (0.57–1.75)	0.992	0.69 (0.37–1.30)	0.255	0.99 (0.53–1.85)	0.983	1.17 (0.63–2.17)	0.624	2.03 (0.81–5.09)	0.131
Cough	1.64 (0.79–3.43)	0.184	1.80 (1.03–3.14)	0.038	1.42 (0.76–2.64)	0.272	1.91 (1.03–3.54)	0.040	1.71 (0.93–3.14)	0.084	1.42 (0.58–3.48)	0.438
Dyspnea	1.07 (0.49–2.35)	0.863	2.53 (1.36–4.72)	0.004	1.92 (0.96–3.82)	0.065	3.24 (1.59–6.58)	0.001	2.47 (1.25–4.89)	0.009	1.35 (0.50–3.68)	0.555
Diarrhea	2.07 (0.88–4.87)	0.097	1.97 (1.08–3.61)	0.028	1.01 (0.52–1.99)	0.968	1.81 (0.92–3.55)	0.085	1.82 (0.94–3.55)	0.077	1.13 (0.43–2.96)	0.806
Anosmia	11.14 (3.92–31.67)	<0.001	0.72 (0.41–1.27)	0.259	1.52 (0.80–2.91)	0.203	0.92 (0.49–1.71)	0.784	0.83 (0.45–1.53)	0.549	10.57 (2.88–38.80)	<0.001
Taste loss	5.50 (2.23–13.56)	<0.001	0.96 (0.54–1.68)	0.877	1.38 (0.72–2.61)	0.330	1.40 (0.75–2.63)	0.291	1.27 (0.68–2.36)	0.449	3.81 (1.35–10.75)	0.011

^§^ In these logistic regression models, serum antibody levels were converted to a binary dependent variable, based on the median value of the sample (0: ≤median, 1: >median). Median values were: 5.47 for anti-S1–IgG–Euroimmun; 53,799 for anti-N; 45,036 for anti-S1 (multi-ELISA); and 42,753 for anti-RBD (multi-ELISA). MFI: mean fluorescence intensity; OD: optical density.

## Supplementary Materials

Supplementary Materials can be found at https://www.mdpi.com/2076-2607/8/12/1885/s1.
