# Supplementary material for: Anti–SARS-CoV-2 Antibody Responses in Convalescent Plasma Donors Are Increased in Hospitalized Patients; Subanalyses of a Phase 2 Clinical Study
Source: Microorganisms. 2020 Nov 28;8(12):1885. doi: 10.3390/microorganisms8121885 (PMC7760522; doi:10.3390/microorganisms8121885)
Supplement: Supplementary file 1 [file microorganisms-08-01885-s001.pdf]

Supplementary File

Anti–SARS-CoV-2 Antibody Responses in Convalescent Plasma Donors Are Increased in Hospitalized Patients: Sub-analyses of a Phase 2 Clinical Study

E. Terpos et al.

Inclusion criteria for the plasma donors: (i) signed informed consent; (ii) confirmed SARS-CoV-2 infection by PCR of the nasal and/or pharyngeal swab; (iii) interval of at least 14 days after complete recovery from a SARS-CoV-2 infection (no symptoms, complete resolution of organ dysfunction which was caused by SARS-CoV-2); (iv) anti-SARS-CoV-2 immune response with anti-SARS-CoV-2 antibodies; (v) two negative SARS-CoV-2 PCR results (nasal and/or pharyngeal swap); the time interval between the two negative tests should be at least 7 days; (vi) the second negative test should be at least 7 days prior to the plasmapheresis; (vii) male donors without transfusion history and females without history of transfusion or pregnancy; (viii) normal full blood count; (ix) negative serological tests for HBV, HCV, HIV, VDRL, HTLV-1 and negative for HIV, HBV, HCV (with NAT); (x) donors should fulfill the general criteria for blood donation: age 18-60 for first time donors, age up to 65 for multiple times donors, body weight >=50 kg, good general condition, Hb >12.5 g/dL for female, Hb >13.5 g/dL for male donors; (xi) Total serum protein ≥60 g/l; (xii) normal IgG levels; (xiii) arterial blood pressure 100-180 mmHg, diastolic<100 mmHg; (xiv) heart rate 50-110/min (for athletes a heart rate <50/min was accepted); (xv) normal body temperature.

Data collection for plasma donors: In order to correlate the level of anti-SARS-CoV-2 antibodies to the severity of the disease the following donor information was collected: (i) time of initial symptoms of COVID-19 infection; (ii) time of positive PCR result of nasal/pharyngeal swab for SARS-CoV-2; (iii) disease symptoms (fever, fatigue, diarrhea, dyspnea, loss of smell, loss of taste, etc); (iv) organs involved in the infection as determined by clinical examination, laboratory or imaging tests (i.e. lung CT scan); (v) time from diagnosis to full recovery of symptoms of the disease; (vi) time from diagnosis to the first and second negative PCR result of nasal/pharyngeal swab for SARS-CoV-2; (vii) need for hospitalization during COVID-19 infection; (viii) need for oxygen supply during the infection; (ix) need for ICU admission or for mechanical ventilation.

RNA Extraction and Real Time RT-PCR for SARS-CoV-2: Please see supplementary file Nasopharyngeal and/or oropharyngeal swabs were collected and transferred to the Microbiology laboratory, immersed in an appropriate virus transport medium (e.g. UTM Viral Transport, Copan Diagnostics Inc., Brescia, Italy). Automated purification of viral RNA from viral transport medium was performed using the QIAsymphony DSP virus/pathogen mini kit on the QIAsymphony SP platform (QIAGEN, Hilden, Germany). A Real Time, one – step Reverse Transcription – PCR, specific for ORF1ab gene of SARS-CoV-2 is finally performed using the VIASURE SARS-CoV-2 Real Time PCR Detection Kit (CerTestBiotec SL, Zaragoza, Spain).

Detection of anti-SARS-CoV2 antibodies using Euroimmun Elisa: Both assays for IgG and IgA antibodies against the S1 domain of the virus were performed on the automated EUROIMMUN Analyzer I (Euroimmun Medizinischem Labordiagnostika AG, Lubeck, Germany), according to the manufacturer's protocol. The anti-SARS-CoV-2 IgG ELISA has 90% sensitivity (95% CI: 74.4-96.5) and 100% specificity (95% CI: 95.4-100), according to manufacturer. The perfromance of the Euroimmun assays for the determination of IgG and IgA antibodies against the SARS-CoV-2 virus was evaluated with sera from patients with COVID-19, hospitalized in Greek hospitals. The infection was confirmed with real time PCR. Only sera collected after the 10th day since the appearance of the symptoms were included. The clinical sensitivity and the 95% CI was 91% (83-97) for the IgG and 97.5% (93-99) for the IgA assays, respectively. The specificity evaluated with sera collected before 2020 was 97.9% (83-100) for the IgG and 90% (68-98) for the IgA assays, respectively. These performance data have been published pooled with the data of other European COVID-19 laboratories in: Ivo Van Walle, Katrin Leitmeyer, ProfileEeva K Broberg, *The European COVID-19 microbiological laboratories group. Meta-analysis of the clinical performance of commercial SARS-CoV-2 nucleic acid, antigen and antibody tests up to 22 August 2020.*
doi: https://doi.org/10.1101/2020.09.16.20195917 https://www.medrxiv.org/content/10.1101/2020.09.16.20195917v1 (The study has been provisionally accepted for publication in Eurosurveillance).The same sera have been used for the initial evaluation of the Luminex in-house method (see below).

Patients' index values were calculated by dividing patients' sera optical density values (A) by the mean of the duplicate calibrator A values. The results are

interpreted as positive if the index value is >1.1, negative if < 0.9 and borderline between 0.8 and 1.1. Quality control material supplied by the manufacturer was analyzed in each run. Serial analysis of the samples of each patient was performed on the same run in order to have a more accurate comparison between samples.

Detection of anti-SARS-CoV2 antibodies using ProtATonce Multiplex Assay: Details of the assay development and testing can be found at https://www.medrxiv.org/content/10.1101/2020.09.09.20191122v2. In summary, antigens were covalently coupled to color-coded magnetic Luminex microspheres with distinct fluorescent signatures (bead regions) allowing the simultaneous detection of antibody responses to the different antigens from the same serum sample. The SARS-CoV-2 N and S1 antigens were purchased from the Native Antigen Company (Kidlington, UK). All other antigens were from Sino Biological Europe GmbH (Eschborn, Germany). Each sample was incubated with a mixture of the coupled microspheres (Bead Mix) in a well of a 96-well microtiter plate to allow binding of anti-CoV antibodies to the antigens. Any unbound material was removed by washing using a magnetic separator. The formed antigen-human anti-CoV antibody complex was incubated with a biotinylated goat anti-human secondary antibody, which binds the specific immunoglobulin type of the anti-CoV human antibodies. Any unbound secondary antibody was removed by a washing step and the formed complex of antigen - human anti-CoV antibody - goat secondary antibody was labelled with streptavidin R-phycoerythrin (SAPE). The fluorescent emission of R-phycoerythrin and the distinct microsphere fluorescent signatures were measured simultaneously by the Luminex® xMAP™-compatible analyzers. The Median Fluorescence Intensity calculated for each antigen was proportional to the amount of anti-CoV antibodies present in the test serum sample. Serum samples were brought to room temperature right before the performance of the assay and diluted 1:400 with sample diluent (except for IgA measurements where a 1:100 dilution was used). A volume of 50 μL from each diluted sample was used for testing. SARS-CoV-2 positive and negative sera generated from a pool of SARS-CoV-2 positive and negative cases, respectively, were used for assay quality control. Results are provided as Median Fluorescence Intensities (MFI) of each antigen across the different samples.

For positive/negative decisions with regard to the presence of antibodies against SARS-CoV-2, cut-off values and decision criteria have been based on the results from

a clinical assay performance study where a total of 155 human serum samples were used; 78 were collected from healthy individuals between 2018-2019 (SARS-CoV-2 negative) and 77 were from PCR confirmed SARS-CoV-2 individuals (SARS-CoV-2 positive). Cut-off MFI values for each SARS-CoV-2 antigen were calculated as the mean MFI of the SARS-CoV-2 negative samples (n=78) + 3 standard deviations (SDs) following removal of outliers. Three decision criteria for each of the three SARS-CoV-2 antigens was based on respective cut-off values. A single decision criterion for antibody presence from N, RBD, and S1 results is based on the optimal logical rule of antigens [RBD OR (S1 AND N)] that is also depicted as a truth table in the Supplementary Table 1This rule was selected from all potential AND/OR combinations in order to optimize the sensitivity, specificity and robustness of the multiplex assay. This rule significantly increases the assay specificity with minimal effect on assay sensitivity as detailed at https://www.medrxiv.org/content/10.1101/2020.09.09.20191122v2.

Detection of anti-SARS-CoV2 antibodies using the in-house Elisa: An in-house ELISA was also developed to detect either the complete Spike (amino acid (AA) 15-1208_2P) or Spike RBD (AA 319-525) using mammalian Expi293-cells produced proteins or E coli produced Nucleocapsid protein spanning the RNA binding domain (AA 47-173). Plates were coated with 0.1 µg of the respective proteins. Eight 4-fold serial dilutions of heat-inactivated sera (starting 1:50) were tested. The antibodies were recognized upon incubation with anti-Human IgG Fc-HRP (Southern Biotech cat#9040-05) and visualized upon incubation with SureBlue TMB substrate (KPL Cat# 5120-0077) and the reaction was stopped with H2SO4. The absorbance was measured at A=450 nm and analyzed by SoftMaxPro software. The area-under-the-curve (AUC) were calculated using GraphPad Prism version 8.0 for MacOS X (GraphPad Software, Inc, La Jolla, CA).

Supplementary Table 1. Decision for positivity based on the results of the multiplex assay

Antigen<br>(+) if higher than cut-off<br>(-) if lower than cut-off			Outcome<br>Positive: SARS-CoV-2 antibodies present<br>Negative: SARS-CoV-2 antibodies absent
N	S1	RBD	
-	-	-	Negative
-	-	+	Negative
-	+	-	Negative
+	-	-	Negative
+	+	-	Negative
+	-	+	Positive
-	+	+	Positive
+	+	+	Positive
